# Item bias detection in the Hospital Anxiety and Depression Scale using structural equation modeling: comparison with other item bias detection methods

**DOI:** 10.1007/s11136-016-1469-1

**Published:** 2016-12-09

**Authors:** Mathilde G. E. Verdam, Frans J. Oort, Mirjam A. G. Sprangers

**Affiliations:** 1 Department of Medical Psychology, Academic Medical Centre, University of Amsterdam, Amsterdam, The Netherlands; 2 Department of Child Development and Education, University of Amsterdam, Postbus 15776, 1001 NG Amsterdam, The Netherlands

**Keywords:** Item bias, Differential item functioning, Structural equation modeling, Hospital Anxiety, Depression Scale

## Abstract

**Purpose:**

Comparison of patient-reported outcomes may be invalidated by the occurrence of item bias, also known as differential item functioning. We show two ways of using structural equation modeling (SEM) to detect item bias: (1) multigroup SEM, which enables the detection of both uniform and nonuniform bias, and (2) multidimensional SEM, which enables the investigation of item bias with respect to several variables simultaneously.

**Method:**

Gender- and age-related bias in the items of the Hospital Anxiety and Depression Scale (HADS; Zigmond and Snaith in Acta Psychiatr Scand 67:361–370, 1983) from a sample of 1068 patients was investigated using the multigroup SEM approach and the multidimensional SEM approach. Results were compared to the results of the ordinal logistic regression, item response theory, and contingency tables methods reported by Cameron et al. (Qual Life Res 23:2883–2888, 2014).

**Results:**

Both SEM approaches identified two items with gender-related bias and two items with age-related bias in the Anxiety subscale, and four items with age-related bias in the Depression subscale. Results from the SEM approaches generally agreed with the results of Cameron et al., although the SEM approaches identified more items as biased.

**Conclusion:**

SEM provides a flexible tool for the investigation of item bias in health-related questionnaires. Multidimensional SEM has practical and statistical advantages over multigroup SEM, and over other item bias detection methods, as it enables item bias detection with respect to multiple variables, of various measurement levels, and with more statistical power, ultimately providing more valid comparisons of patients’ well-being in both research and clinical practice.

**Electronic supplementary material:**

The online version of this article (doi:10.1007/s11136-016-1469-1) contains supplementary material, which is available to authorized users.

## Introduction

Assessment of patient-reported outcomes (PROs) is becoming standard practice in health care and medicine [23]. Implementing PROs into clinical practice helps to understand the impact of illness from the patient’s viewpoint and can make an important contribution to healthcare evaluations [2]. As such, comparing assessments of PROs is becoming increasingly important in both clinical practice and research. However, such comparisons may be invalidated by the occurrence of differential item functioning (DIF). DIF, also referred to as item bias, occurs when two people with the same value on the trait of interest (e.g., well-being) have a different probability of giving a certain response on an item from a questionnaire or test that measures the trait of interest, due to differences on other variables (e.g., age, gender, attitudes, mood, and treatment condition). Mellenbergh [16] gave a formal definition of item bias: An item *X* measuring trait *T* is unbiased with respect to another variable *V*, if and only if:

1 $$f_{ 1} \left( {X|V = v,\;T = t} \right) = f_{ 2} \left( {X|T = t} \right),$$

where *f*_1_ is the distribution of the item responses given the values *v* and *t* of variables *V* and *T*, and *f*_2_ is the distribution of item responses given only the values *t* of variable *T*. Mellenbergh emphasized the generality of the definition, where the variables *X*, *V* and *T* may have nominal, ordinal or interval measurement scales. In the presence of item bias, differences between two people on observed item scores may not reflect “true” differences on the trait variable (e.g., men and women may score differently on an item that measures well-being, even though their well-being does not differ). If the bias is uniform, it is consistent for all levels of the latent trait (e.g., the size of the bias is independent of the level of well-being). When the bias is nonuniform, it differs for different levels of the latent trait (e.g., the difference may be larger for higher levels of well-being).

Statistical methods for the detection of item bias can be distinguished based on their operationalization of the trait variable *T*. One group of methods use the summary of the observed item scores (i.e., the scale score) to operationalize the trait variable (e.g., log-linear models, contingency tables methods, logistic regression models, standardization methods), and another group of methods operationalize an unobserved latent trait variable [e.g., item response theory (IRT) analysis and structural equation modeling (SEM) methods] [17]. We further distinguish between methods that can detect uniform item bias, and methods that can also detect nonuniform item bias. Although advantages have been made to enable the investigation of nonuniform item bias, it is not always easily implemented and therefore not often applied.

Cameron et al. [7] recently investigated the equivalence of three different bias detection methods for the detection of gender- and age-related bias in the items of the Hospital Anxiety and Depression Scale (HADS; [28]). They applied ordinal logistic regression, IRT, and contingency tables methods to investigate item bias in the anxiety and depression subscales of the HADS separately. All three methods were used to detect uniform item bias only. Although Cameron et al. mention SEM methods as a fourth option that can be applied to investigate item bias, they did not incorporate SEM methods in their comparison.

SEM methods may have several important advantages for the detection of item bias. The multigroup SEM approach can be applied to detect bias in observed item scores with respect to group membership (e.g., gender or age category) and a continuous latent trait variable (e.g., depression or anxiety). Advantages of the multigroup SEM approach are that it uses a latent trait operationalization, it enables the detection of both uniform and nonuniform bias, and possible item bias can be taken into account to assess true differences between groups. In addition, the flexibility of the SEM framework allows for an alternative procedure for item bias detection using multidimensional models instead of multigroup models. This enables the investigation of item bias with respect to any factor or variable (e.g., continuous or categorical, latent or manifest). Uniform bias can then be investigated by testing the significance of direct effects of these additional factors on the observed items. However, with multidimensional models the investigation of nonuniform bias is less straightforward and therefore not often applied. Advantages of the multidimensional SEM approach over the multigroup SEM approach are that continuous variables can be included in the model without categorizing them and that item bias can be investigated with respect to several variables simultaneously. Moreover, as it is not necessary to divide the sample into subsamples by group membership, the multidimensional SEM method should also have more statistical power to detect effects.

The objective of the present paper is threefold. First, we illustrate how to apply the multigroup SEM approach to investigate both uniform and nonuniform gender- and age-related item bias in each subscale of the HADS. Second, we illustrate how to apply the multidimensional SEM approach to both subscales of the HADS, and investigate uniform gender- and age-related item bias simultaneously. Third, in order to evaluate possible differences in results between different bias detection methodologies, we compare the results of both SEM approaches to the results of the three item bias detection methods that were investigated by Cameron et al. [7].

## Methods

A total of 1068 adults who consulted a primary care professional in North East Scotland completed the HADS (for more details on data collection see [6]. The HADS is a 14-item self-report instrument that consists of an anxiety (HADS-A; seven items) and depression (HADS-D; seven items) subscale where higher scores represent greater symptom severity. All items are answered on an ordinal response scale with four response categories (0–3). The sample consisted of 435 men and 633 women, with ages ranging between 16 and 92 years (mean age = 50, standard deviation = 18). Mean anxiety scores (HADS-A) were 7.7 with a standard deviation of 4.7, and mean depression scores (HADS-D) were 4.9 with a standard deviation of 4.2.

### Statistical analyses

Structural equation modeling (SEM) was used to investigate gender- and age-related item bias in the anxiety and depression subscales of the HADS. To accommodate discrete ordinal responses, we need to assume that the observed ordinal responses are representations of continuous underlying variables. This enables the estimation of means and variances and covariances, which can be used in subsequent SEM analyses. In addition, alternative estimation methods are needed to yield unbiased parameter estimates and standard errors. These procedures for the analyses of discrete data have been described elsewhere (e.g., [11, 12, 18, 19]). Although different approaches for the investigation of item bias exist, in the present paper we applied the SEM approach for the investigation of bias in discrete ordinal item responses that has been proposed by Verdam et al. [26]. This approach includes two stages: (1) establishing a model of underlying continuous variables that represent the observed discrete variables and (2) using these underlying continuous variables to establish a common factor model for the detection of item bias and to assess true change in the underlying common factors. This SEM approach with discrete data was originally illustrated with longitudinal data, but can also be applied to the multigroup situation. The diagonally weighted least squares (DWLS) estimator with robust standard errors was used to yield unbiased parameter estimates and precise standard errors (e.g., [9, 10, 27]). The weighted least squares (WLS) Chi-square value was used for the evaluation of model fit, as it follows an asymptotic Chi-square distribution (if the model holds) and can therefore also be used for the calculation of differences in model fit and approximate fit indices (see [26] for more details). Statistical analyses were performed using the PRELIS (Stage 1) and LISREL (Stage 2) programs [15]. Syntax files for reported analyses are available in “Appendix A” of supplementary material (Stage 1) and “Appendix B” of supplementary material (Stage 2).

### Multigroup SEM procedure

**Fig. 1:**
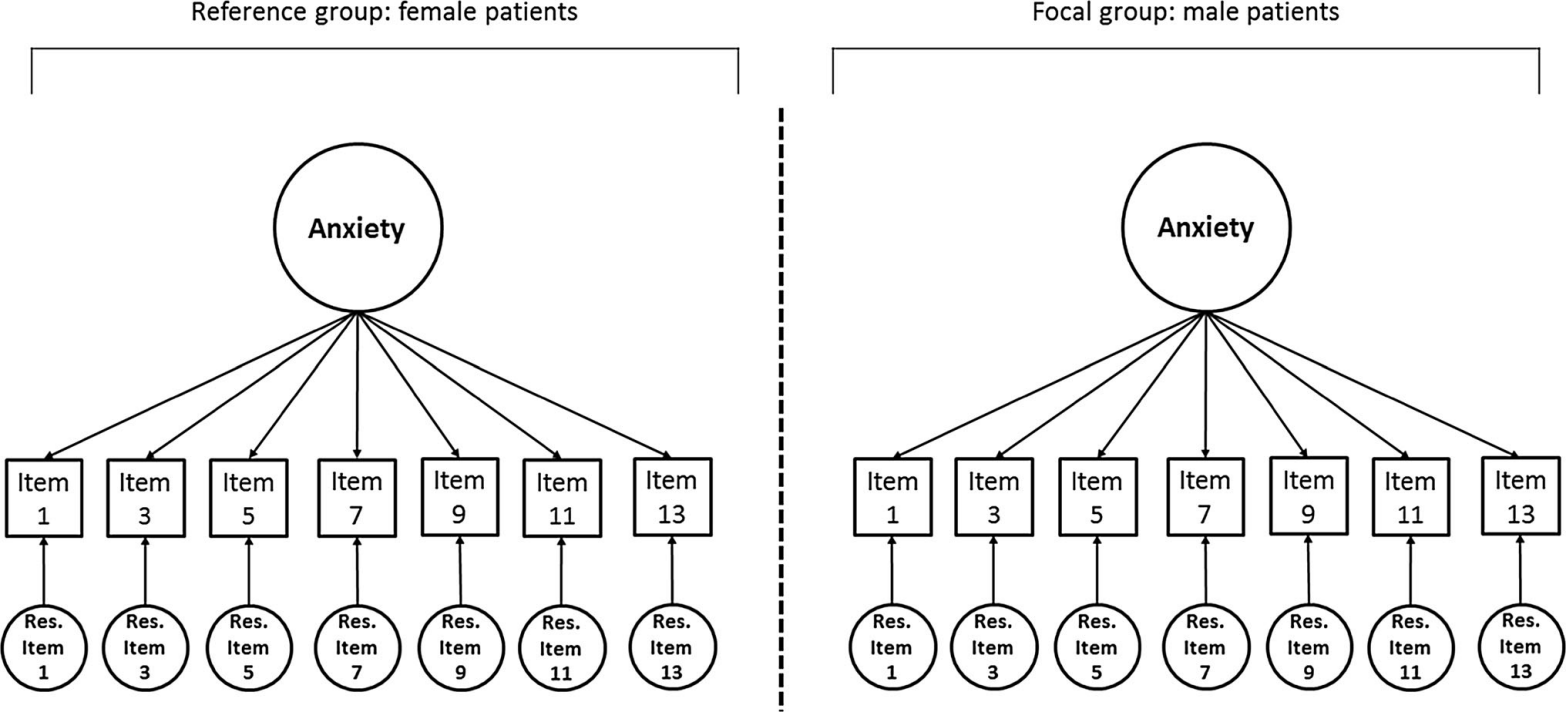
Two-group Measurement Model for gender-related item bias detection in the anxiety subscale of the HADS. Similar models have been used for the detection of age-related item bias in the anxiety subscale of the HADS, and for the detection of gender- and age-related item bias in the depression subscale of the HADS. The squares represent the underlying continuous variables associated with the observed item responses of Item 1 to Item 13. The circle at the top is the underlying common factor Anxiety, which represents everything that Item 1 to Item 13 have in common. Each item is associated with a residual factor, which represents everything that is specific to the corresponding item. Item bias is operationalized as across-group differences in intercepts (uniform) and factor loadings (nonuniform)

Gender- and age-related item bias was investigated for the anxiety and depression subscales of the HADS separately, by comparing a “reference” and “focal” group. For age, there were 814 participants in the reference group (<65 years) and 254 participants in the focal group (>65 years). For gender, there were 633 participants in the reference group (women) and 435 in the focal group (men). The categorization of age and the separate analysis of the subscales of the HADS were chosen in order to enable comparison of the SEM results with the results from the other detection methods as reported by Cameron et al. [7]. Figure 1 gives a graphical representation of the multigroup model for item bias detection.

In Stage 1, the model of underlying continuous variables that represent the observed discrete variables was used to estimate thresholds and polychoric correlations under the assumption of bivariate normality in both groups. Thresholds of the same items were constrained to be equal across groups. The tenability of the assumption of underlying bivariate normality in each group was evaluated using the root mean square error of approximation (RMSEA; [24, 25]), with the criterion that RMSEA values should not be larger than 0.1 [13]. When the hypothesis of bivariate normality under equal thresholds holds for all pairs of variables, the estimated polychoric correlations, variances, and means of the underlying continuous variables can be used in subsequent analyses of Stage 2. When the hypothesis of bivariate normality does not hold, then this indicates that the assumption of multivariate normality (under equal thresholds) is not tenable. A possible solution for this problem is to eliminate the offending variable(s).

In Stage 2, *Step 1*, the estimates from the underlying variables from Stage 1 were used to establish a multigroup common factor model (e.g., a one-factor model for “Anxiety,” with seven indicator items, for both men and women; see Fig. 1). The Measurement Model has no across-group constraints. The appropriateness of the Measurement Model was evaluated using overall goodness of fit. The Chi-square test can be used to evaluate exact goodness of fit, where a significant Chi-square value indicates a significant difference between data and model. However, in the practice of SEM exact fit is rare, and with large sample sizes or parsimonious models the Chi-square test generally turns out to be significant. Therefore, as an alternative, we used the RMSEA value as a measure of approximate goodness of fit, where values below .08 indicate “reasonable” approximate fit and below .05 “close” approximate fit [5]. Many other approximate fit indices have been proposed for the evaluation of overall model fit, such as the comparative fit index [4] and Akaike’s [1] information criterion, but all these indices are derived from the same discrepancy function, just as the Chi-square statistic and the RMSEA. In the present study, these other fit indices do not provide additional information, and we concisely used the RMSEA as the only overall model fit criterion.

In *Step 2*, the No Item Bias Model was fitted to the data, where all measurement parameters were constrained to be equal across groups. Item bias was operationalized as across-group differences between values of intercepts (i.e., uniform item bias; across-group differences in the endorsement of an item, independent from the latent trait variable) and differences between common factor loadings (i.e., nonuniform item bias; across-group differences in the extent to which an item measures the latent trait variable). Differences between residual variances are not considered in the present paper, as they do not affect the assessment of true differences. To test for the presence of item bias, the No Item Bias Model can be compared to the Measurement Model. The Chi-square difference test was used to test the difference in exact fit, where a significant Chi-square difference indicates that the No Item Bias Model has significantly worse fit as compared to the Measurement Model. If the invariance restrictions of the No Item Bias Model led to a significant deterioration in model fit, this indicated the presence of item bias. As the Chi-square test statistic is very sensitive to large sample sizes, to guard against false positives, we considered *p* values <.001 to indicate statistical significance, similar to Cameron et al. [7].

In case of item bias, in *Step 3*, a step-by-step modification of the No Item Bias Model was used to arrive at the Final Model in which all items that showed item bias were taken into account. The identification of item bias was guided by an iterative procedure, where each across-group constraint was set free one at a time, and the freely estimated parameter that led to the largest improvement in fit was included in the model. Each indication of bias was tested by evaluating the improvement in model fit using the Chi-square difference test to evaluate differences in exact fit. To guard against false positives, we considered *p* values <.001 to indicate statistical significance. The Final Model was compared to the Measurement Model to test equivalence of exact fit as an indication that all apparent item bias was taken into account. To give an indication of the size of the detected item bias, we calculated Cohen’s *d* effect size indices for the impact of both uniform and nonuniform item bias on the differences between the item means across groups (see [22] for more details), where values of 0.2, 0.5, and 0.8 indicate small, medium, and large effects [8]. Following the example of Cameron et al. [7], we used importance criteria in addition to significance criteria for the detected item bias (see also Table 2), where item bias was considered “important” when the size of the item bias was larger than 0.2.

In *Step 4*, the estimates of common factor means of the Final Model, in which all apparent item bias was taken into account, was used to assess true differences between the groups. Cohen’s *d* effect size was calculated to give an indication of the size of the difference. In addition, the overall impact of item bias on the assessment of true differences can be evaluated through the comparison of effect size indices before and after taking possible item bias into account.

### Multidimensional SEM procedure

**Fig. 2:**
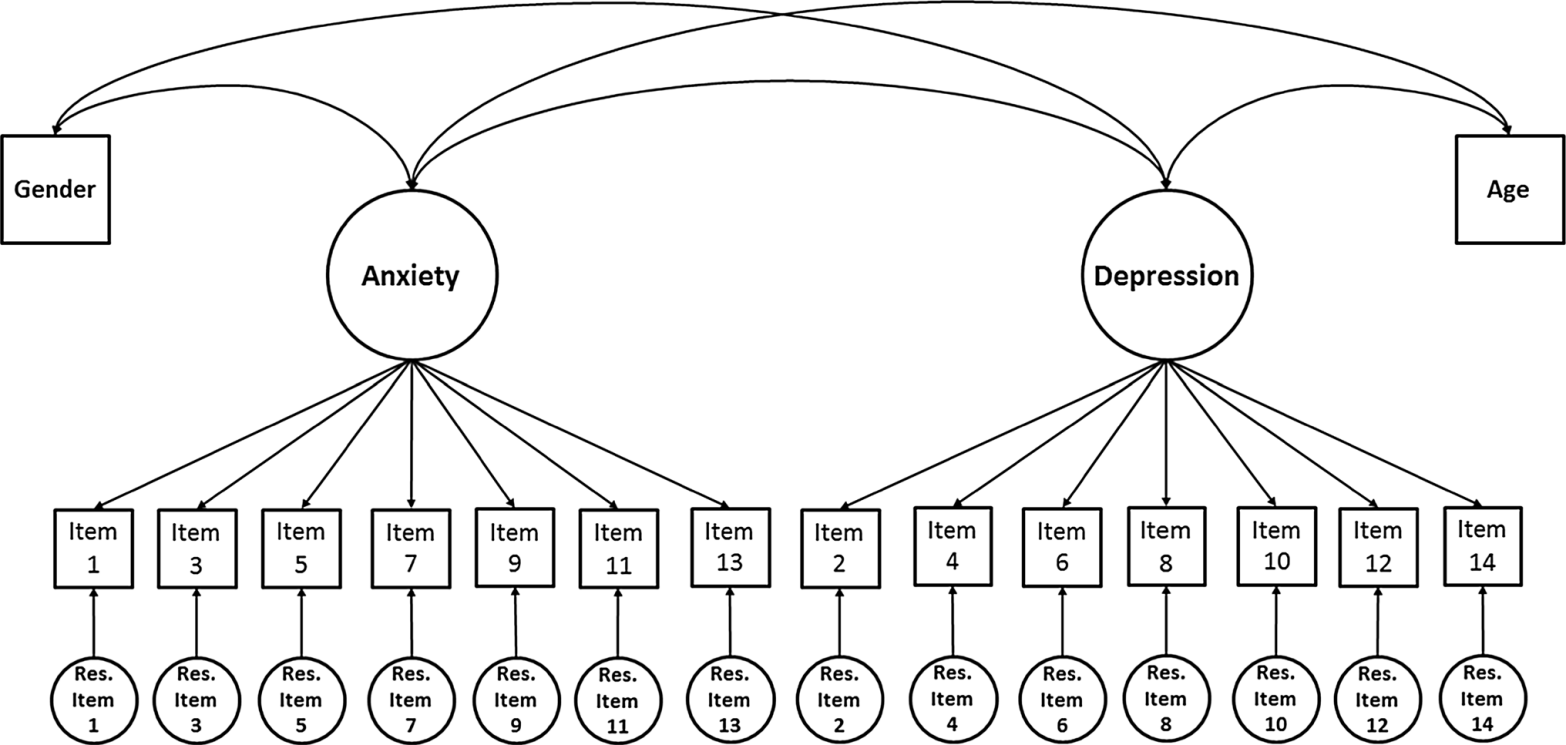
Multidimensional “no item bias” model for gender- and age-related item bias detection in the anxiety and depression subscales of the HADS. The squares represent the underlying continuous variables associated with the observed item responses of Item 1 to Item 14. The circles at the top are the underlying common factors Anxiety and Depression. Anxiety represents everything that Item 1 to Item 13 have in common, whereas Depression represents everything that Item 2 to Item 14 have in common. Each item is associated with a residual factor, which represents everything that is specific to the corresponding item. The multidimensional model includes two exogenous variables: Gender and Age. Uniform item bias is operationalized as significant direct effects of the exogenous variables on the indicator variables (i.e., Item 1 to Item 14)

Gender- and age-related item bias was investigated for the anxiety and depression subscales of the HADS simultaneously, by including both age and gender as exogenous variables in the multidimensional model. Figure 2 gives a graphical representation of the multidimensional model for item bias detection. The multidimensional SEM procedure that is used in the present article is also known as the restricted factor analysis (RFA) procedure as originally described by Oort [20, 21]. It yields equivalent results as multiple-indicator multiple-cause (MIMIC; [14]) analysis, but in MIMIC the associations between the violators (e.g., age and gender) and the constructs of interest (e.g., depression and anxiety) are modeled through causal relations, whereas in RFA they are modeled through correlations. The procedure for item bias detection using the multidimensional approach was largely similar to the procedure for item bias detection using the multigroup approach. Here, we describe only the differences in the procedures. In Stage 1, correlations between all variables in the model (i.e., the underlying variables that correspond to the observed items and the exogenous variables) were estimated. In Stage 2, *Step 1*, the estimates from the underlying variables from Stage 1 were used to establish a multidimensional Measurement Model that included the common factors “Anxiety” and “Depression,” each with seven indicator variables. In *Step 2*, the multidimensional Measurement Model was extended to include the variables “Age” and “Gender.” These variables were allowed to correlate with the common factors, but all direct effects of Age and Gender on the items were constrained to zero. This model is referred to as the No Item Bias Model. The overall model fit of this model was used to give an indication of the presence of item bias, where an RMSEA value <.08 was taken as a global indication that there was no presence of item bias. In *Step 3*, an iterative procedure was used, where each constrained direct effect of the exogenous variables age and gender was set free to be estimated one at a time, and the freely estimated parameter that led to the largest improvement in fit according to the Chi-square difference test was included in the model, where *p* < .001 was taken to indicate statistical significance. When freeing additional parameters did not lead to a significant improvement in model fit, this was taken as an indication that all apparent bias was taken into account. The importance criterion for item bias was evaluated using the standardized direct effects, which can be interpreted as effect size *r*, with values of 0.1, 0.3, and 0.5 indicating small, medium, and large effect sizes [8]. In *Step 4*, the correlations between the exogenous variables age and gender and the common factors of the Final Model, in which all apparent bias has been taken into account, were used to assess true differences between the genders, and true associations with age. The overall impact of item bias on the assessment of true differences between the genders and true associations with age can be evaluated through the comparison of correlations before and after taking possible item bias into account.

## Results

Model fit results of the item bias detection procedures are presented in Table 1. An overview of the items that were identified as having bias by either SEM approach and the item bias detection results from Cameron et al. [7] are given in Table 2. We first report results of the multigroup SEM approach and then those of the multidimensional SEM approach. Subsequently, we compare the results of both SEM approaches to the results from the bias detection methods reported by Cameron et al.

**Table 1:** Goodness of overall model fit and difference in model fit of the models for gender- and age-related item bias detection models in Stage 2; for both the multigroup structural equation modeling approach, and the multidimensional structural equation modeling approach

Model		*Df*	CHISQ	*p* value	RMSEA [90% CI]	Compared to	*Df*_diff_	CHISQ_diff_	*p* value
*Multigroup gender*-*related item bias detection*									
Anxiety subscale									
1a	Measurement Model	28	50.64	.005	0.039 [0.021; 0.056]				
1b	No Item Bias Model	40	126.4	<.001	0.064 [0.051; 0.076]	Model 1a	12	75.76	<.001
1c	Final Model	38	81.29	<.001	0.046 [0.032; 0.060]	Model 1a	10	30.65	<.001
Depression subscale									
2a	Measurement Model	28	46.26	.016	0.035 [0.015; 0.052]				
2b	No Item Bias Model	40	120.9	<.001	0.062 [0.049; 0.074]	Model 2a	12	74.63	<.001
2c	Final Model	37	70.02	<.001	0.041 [0.026; 0.055]	Model 2a	9	23.76	.005
*Multigroup age*-*related item bias detection*									
Anxiety subscale									
3a	Measurement Model	28	61.02	<.001	0.047 [0.031; 0.063]				
3b	No Item Bias Model	40	163.2	<.001	0.076 [0.064; 0.088]	Model 3a	12	102.1	<.001
3c	Final Model	37	81.71	<.001	0.048 [0.04; 0.062]	Model 3a	9	20.69	.014
Depression subscale									
4a	Measurement Model	28	42.59	.038	0.031 [0.008; 0.049]				
4b	No Item Bias Model	40	357.2	<.001	0.122 [0.111; 0.134]	Model 4a	12	314.6	<.001
4c	Final Model	34	83.24	<.001	0.052 [0.038; 0.066]	Model 4a	6	40.65	<.001
*Multidimensional gender*- *and age*-*related item bias detection*									
Anxiety and Depression subscale									
5a	Measurement Model	76	485.05	<.001	0.071 [0.065; 0.077]				
5b	No Item Bias Model	100	1029.8	<.001	0.093 [0.088; 0.098]				
5c	Final Model	88	455.71	<.001	0.063 [0.057; 0.068]				

*N* = 1068. Overall model fit and difference in fit was evaluated using WLS Chi-square values that are provided in the standard LISREL output (denoted C2_NNT)

**Table 2:** Results of gender- and age-related item bias detection in the anxiety and depression scales of the HADS questionnaire using the multigroup structural equation modeling (SEM-MG) and multidimensional structural equation modeling (SEM-MD) approaches

Item	Gender-related item bias					Age-related item bias				
	LOGR^1^	IRT^2^	CONT^3^	SEM-MG^4^	SEM-MD^5^	LOGR^1^	IRT^2^	CONT^3^	SEM-MG^4^	SEM-MD^5^
*HADS*-*A*										
1. I feel tense or wound up	–	–	–	–	–	−**0.77** ^a^	**−0.61** ^a^	**−3.78** ^a^	**−0.22** ^**a**^	−*0.09* ^a^
3. I get a … feeling as if something awful…	–	–	–	–	–	–	–	–	*0.09* ^b^	–
5. Worrying thoughts go through my mind	–	–	–	–	–	–	–	–	–	–
7. I can sit at ease and feel relaxed	–	–	–	–	–	–	–	–	–	–
9. I get a.. feeling like ‘butterflies’ in the stomach	−*0.49* ^a^	–	**−3.64** ^a^	−*0.16* ^a^	**−0.13** ^a^	–	–	–	–	–
11. I feel restless as if I have to be on the move	*0.58* ^a^	**−0.62** ^a^	**4.70** ^a^	**0.26** ^a^	**0.12** ^a^	–	–	–	–	–
13. I get sudden feelings of panic	–	–	–	–	–	–	–	–	**0.22** ^a^	*0.07* ^a^
*HADS*-*D*										
2. I still enjoy the things I used to enjoy	–	–	–	–	*0.07* ^a^	–	–	–	–	–
4. I can laugh and see the funny side of things	–	–	–	**−0.20** ^a^ *0.01* ^b^	–	–	–	–	**−0.66** ^**a**^ *0.01* ^b^	**−0.13** ^a^
6. I feel cheerful	–	–	–	–	**0.11** ^a^	**−1.11** ^a^	**−0.77** ^a^	**-5.16** ^a^	**−0.56** ^**a**^	**−0.23** ^a^
8. I feel as if I am slowed down	–	–	–	–	–	**0.92** ^a^	**1.03** ^a^	**6.72** ^a^	–	**0.14** ^a^
10. I have lost interest in my appearance	–	–	–	**−** *0.01* ^b^	–	**−** *0.60* ^a^	**−0.52** ^a^	**−3.66** ^a^	**−0.34** ^**a**^	**−0.14** ^a^
12. I look forward with enjoyment to things	–	–	–	–	–	–	–	–	**−** *0.01* ^b^	–
14. I can enjoy… book or radio or TV	–	–	–	–	**0.12** ^a^	–	–	–	**−0.29** ^**a**^	**−0.18** ^a^

Results are compared to the item bias detection results as reported by Cameron et al. [7] from the ordinal logistic regression method (LOGR), the item response theory method (IRT), and the contingency table method (CONT)

^a^Uniform item bias

^b^Nonuniform item bias. Results meeting the criteria for important item bias are marked in bold, results meeting only the significance criterion are marked in italics. Numbers are given only for those item bias detection results that were considered statistically significant

^1^Log odds ratios are presented, where items were regarded as having important bias if the absolute magnitude of the log odds ratio was greater than 0.64 and *p* < 0.001

^2^Contrasts with absolute values greater than 0.50 and *p* < 0.05 were taken as an indication of important item bias

^3^Standardized Liu–Agresti cumulative common log odds ratios (LOR Z) are presented, where absolute values <2 and *p* < .001 are considered important item bias

^4^Effect size indices *d* are presented. For uniform item bias, these refer to the difference in intercept parameters between the groups, divided by the pooled standard deviation. For nonuniform item bias these refer to the difference in factor loading parameter multiplied with the difference in common factor means between the groups, divided by the pooled standard deviation. Effect sizes larger than .20 and *p* < .001 are indicative of important item bias

^5^Effect size indices *r* are presented, which are the standardized direct effect of Gender/Age on the specific item. Effect sizes larger than .10 and *p* < .001 are indicative of important item bias

### Multigroup SEM approach

Results of Stage 1 indicated that the hypothesis of bivariate normality under equal thresholds was tenable for all item pairs, for both subscales and both gender and age groups. Estimated polychoric correlations, variances, and means were used in subsequent analyses of Stage 2. We report results of gender- and age-related item bias for each subscale of the HADS separately.

#### Anxiety subscale

##### Gender-related item bias

Results of Stage 2 indicated that the Measurement Model showed close approximate fit (Model 1a, Table 1). Imposition of equality constraints on measurement parameters across groups yielded the No Item Bias Model (Model 1b). The No Item Bias Model showed a significant deterioration in model fit as compared to the Measurement Model, indicating the presence of gender-related item bias of the HADS-A (see Table 1). Indications of uniform bias were detected for Item 9 (CHISQ_diff_ (1) = 14.54, *p* < .001) and for Item 11 (CHISQ_diff_ (1) = 30.57, *p* < .001). The Final Model, in which both biases were incorporated in the model, showed close approximate fit (Model 1c, Table 1). Although the Final Model did not yield equivalent fit as compared to the Measurement Model, freeing additional parameters did not significantly improve model fit.

##### Age-related item bias

The Measurement Model showed close approximate fit (Model 3a, Table 1). The No Item Bias Model yielded a significant deterioration in model fit, indicating the presence of age-related item bias of the HADS-A. Two items with uniform bias and one item with nonuniform bias were identified. The Final Model that incorporated these three biases (Model 3c) showed equivalent fit compared to the Measurement Model (see Table 1). Uniform bias was detected for Item 1 (CHISQ_diff_ (1) = 18.36, *p* < .001) and for Item 13 (CHISQ_diff_ (1) = 50.78, *p* < .001), whereas nonuniform bias was detected for Item 3 (CHISQ_diff_ (1) = 12.31, *p* < .001).

##### True differences between the groups

Inspection of common factor means showed that men score significantly lower on the Anxiety factor as compared to women (*d* = −0.30, *p* < .001) and that patients older than 65 scored significantly lower on the Anxiety factor compared to patients younger than 65 (*d* = −0.76, *p* < .001). If item bias would not have been taken into account the true differences between the gender and age groups would have been estimated to be similar (*d* = −0.26, *p* < .001; and *d* = −0.73, *p* < .001, respectively).

#### The depression subscale

##### Gender-related item bias

The Measurement Model indicated close approximate fit (Model 2a, Table 1). Comparison of the No Item Bias Model with the Measurement Model indicated the presence of gender-related item bias of the HADS-D. Step-by-step modification of the No Item Bias Model yielded the Final Model in which all bias was taken into account (Model 2c, Table 1). For Item 4, both uniform bias [CHISQ_diff_ (1) = 16.55, *p* < .001] and nonuniform bias were detected [CHISQ_diff_ (1) = 14.47, *p* < .001]. In addition, nonuniform bias was detected for Item 10 [CHISQ_diff_ (1) = 18.85, *p* < .001]. The Final Model showed equivalent fit as compared to the Measurement Model (see Table 1).

##### Age-related item bias

The Measurement Model showed close approximate fit (Model 4a, Table 1), but comparison with the No Item Bias Model indicated the presence of age-related item bias of the HADS-D (see Table 1). Uniform bias was detected in four items, and nonuniform bias was detected in three items, where one item showed both uniform and nonuniform bias. The Final Model, which included all apparent bias, showed close approximate fit (Model 4c). Although the Final Model did not yield equivalent fit as compared to the Measurement Model (see Table 1), freeing additional parameters did not significantly improve model fit. Uniform bias was detected for Item 4 [CHISQ_diff_ (1) = 63.68, *p* < .001], Item 6 [CHISQ_diff_ (1) = 102.97, *p* < .001), Item 10 [CHISQ_diff_ (1) = 40.12, *p* < .001], and Item 14 [CHISQ_diff_ (1) = 30.57, *p* < .001]. Nonuniform bias was detected for Item 4 [CHISQ_diff_ (1) = 16.06, *p* < .001] and Item 12 [CHISQ_diff_ (1) = 20.51, *p* < .001].

##### True differences between the groups

There were no significant differences between men and women (*d* = 0.03, *p* = .64) or between the age groups (*d* = 0.03, *p* = .70) with respect to their scores on the underlying Depression factor. Before taking into account item bias true differences between men and women were estimated to be similar (*d* = −0.01, *p* = .88). However, true differences between the age groups were estimated to be negative and significant (*d* = −0.34, *p* < .001). Thus, if item bias would not have been taken into account the difference in depression severity between the age groups would have been overestimated.

### Multidimensional SEM approach

Results of Stage 1 indicated that the hypothesis of bivariate normality under equal thresholds was tenable for all combinations of items and exogenous variables. The estimated (polychoric) correlations, variances, and means of all variables were used for subsequent analyses in Stage 2. In Stage 2, the Measurement Model that included both HADS subscales showed reasonable approximate fit (Model 5a, Table 1). The No Item Bias Model that included the variables Age and Gender did not show acceptable fit (Model 5b), indicating the presence of item bias (see Table 1). Uniform bias was detected in four items of the HADS-A, and six items of the HADS-D. The Final Model, which included all apparent bias, showed reasonable approximate fit (Model 5c, Table 1).

#### The anxiety subscale

Gender-related bias of the HADS-A was detected for Item 9 [CHISQ_diff_ (1) = 24.2, *p* < .001] and Item 11 [CHISQ_diff_ (1) = 97.9, *p* < .001]. Age-related bias of the HADS-A was detected for Item 1 [CHISQ_diff_ (1) = 64.0, *p* < .001] and Item 13 [CHISQ_diff_ (1) = 104.8, *p* < .001].

#### The depression subscale

Gender-related bias of the HADS-D was detected for Item 2 [CHISQ_diff_ (1) = 22.9, *p* < .001], Item 6 [CHISQ_diff_ (1) = 28.2, *p* < .001], and Item 14 [CHISQ_diff_ (1) = 28.9, *p* < .001]. Age-related bias of the HADS-D was detected for Item 4 [CHISQ_diff_ (1) = 25.9, *p* < .001], Item 6 [CHISQ_diff_ (1) = 66.4, *p* < .001], Item 8 [CHISQ_diff_ (1) = 20.8, *p* < .001], Item 10 [CHISQ_diff_ (1) = 37.6, *p* < .001], and Item 14 [CHISQ_diff_ (1) = 52.5, *p* < .001].

#### True differences and associations

Inspection of parameter estimates of the Final Model showed that there was a significant positive association between Anxiety and Depression (*r* = 0.83, *p* < .001), indicating that symptom severity with respect to Anxiety goes together with symptom severity with respect to Depression. There was a significant negative association between Age and Anxiety (*r* = −0.24, *p* < .001), indicating that older patients scored lower on Anxiety than younger patients. There was also a significant negative association between Gender and Anxiety (*r* = −0.16, *p* < .001), indicating that men scored lower on Anxiety than women. The association between Gender and Depression was negative, and between Age and Depression was positive, but neither was significant (*r* = −0.04, *p* = .19, and *r* = 0.01, *p* = .83, respectively). Lastly, there was a significant positive association between Age and Gender (*r* = 0.11, *p* < .001), indicating that men were—on average—significantly older than women. If item bias would not have been taken into account, the pattern and size of true differences and associations would have been estimated to be similar, with the exception of the association between Age and Depression. Without taking into account item bias this association was estimated to be negative and significant (*r* = −0.10, *p* < .001).

### Comparison with results from the ordinal logistic regression, item response theory, and contingency table methods

With regard to the anxiety subscale of the HADS, both the multigroup SEM approach and multidimensional SEM approach identified uniform gender-related bias in Item 9 (“I get a … feeling like ‘butterflies’ in the stomach”) and Item 11 (“I feel restless as if I have to be on the move”). These detected biases indicate that anxiety symptoms manifested themselves differently in men as compared to women, where restlessness was more prevalent in men and “butterflies” in the stomach were more prevalent in women, relative to the level of anxiety. These results are largely consistent with the results from Cameron et al., as the contingency tables method and the ordinal logistic regression method identified the same items as biased, with similar size and direction of detected bias. The IRT method only detected uniform gender-related bias in Item 11, where the result was in the opposite direction.

With regard to the anxiety subscale of the HADS, both the multigroup SEM approach and multidimensional SEM approach identified uniform age-related bias in Item 1 (“I feel tense or wound up”) and Item 13 (“I get sudden feelings of panic”). Taking into account the reversed scoring of the contraindicative items, the detected biases indicate that patients older than 65, as compared to patients younger than 65, experienced more symptoms of panic and tenseness, relative to the level of anxiety. All methods reported by Cameron et al. also identified age-related bias in Item 1. The (small) uniform age-related bias of Item 13 was not detected by the methods of Cameron et al., although the results of the contingency tables method and ordinal logistic regression method almost reached statistical significance for this item (*p* = .001 for both methods). In addition to the detected uniform biases, the multigroup SEM approach also detected nonuniform age-related bias in Item 3.

With regard to the depression subscale, the methods reported by Cameron et al. did not detect gender-related item bias, whereas both SEM methods did detect gender-related bias. The multigroup SEM approach detected uniform bias in Item 4 (“I can laugh and see the funny side of things”), whereas the multidimensional SEM approach detected uniform bias in Item 6 (“I feel cheerful”) and Item 14 (“I can enjoy… book or radio or TV”). These results indicate that men, as compared to women, reported to be less able to see the funny side of things, but experienced more cheerful feelings and enjoyment with a book/radio/TV, relative to the level of anxiety. In addition, the multidimensional SEM approach identified a small uniform bias in Item 2, and the multigroup SEM approach identified small nonuniform bias in Items 4 and 10. As the results of uniform bias detection were not consistent across both SEM methods and were not confirmed by the methods applied by Cameron et al., they should be interpreted with caution.

All three methods reported by Cameron et al. detected age-related bias in Item 6 (“I feel cheerful”), Item 8 (“I feel as if I am slowed down”), and Item 10 (“I have lost interest in my appearance”) of the depression subscale. The results of the multigroup SEM approach confirmed the age-related bias in Items 6 and 10, and the results from the multidimensional SEM approach confirmed the age-related bias in all three items. Taking into account reversed scoring of contraindicative items, these results indicate that patients older than 65, as compared to patients younger than 65, indicated to be more cheerful, but also that they were more slowed down, whereas they indicated to lose less interest in their appearance, relative to the level of depression. The SEM approaches also identified additional items with uniform and nonuniform age-related bias in the depression subscale. Both SEM methods detected uniform bias in Item 4 (“I can laugh and see the funny side of things”) and Item 14 (“I can enjoy … book or radio or TV”). These results indicate that patients older than 65, as compared to patients younger than 65, indicated to see the funny side of things and enjoy a book more, relative to the level of depression. In addition to the detected uniform biases, the multigroup SEM approach also detected nonuniform age-related bias in Items 4 and 12.

## Discussion

We illustrated how to apply two different SEM methods for the detection of gender- and age-related item bias in the anxiety and depression subscales of the HADS, to account for item bias, and to more validly evaluate patients’ anxiety and depression. Specifically, we used a multigroup SEM approach to investigate both uniform and nonuniform item bias in each subscale of the HADS separately, and a multidimensional SEM approach that enabled the investigation of uniform item bias in both subscales of the HADS and with regard to both gender and age simultaneously. Results from the multigroup SEM approach and the multidimensional SEM approach with regard to the detection of uniform item bias were largely consistent, and generally agreed with the results of the ordinal logistic regression, item response theory (IRT), and contingency tables methods reported by Cameron et al. [7] as the same items were identified as biased. However, the SEM approaches also identified additional items with bias. Below, we first discuss the results of both SEM approaches, and subsequently discuss the difference between the results of both SEM approaches and the results from the other bias detection methods.

The multigroup SEM method identified a total of ten items with bias, of which eight items showed uniform bias and four items showed nonuniform bias. The multidimensional SEM method was used to detect only uniform bias and identified a total of ten items as biased. These indications of bias may invalidate the comparison of item scores for men and women, and subjects with different ages or from different age groups. However, the overall effect of detected item biases on the assessment of true differences in and associations with anxiety and depression severity was generally small. Only for the depression subscale of the HADS the detected item bias would have led to an overestimation of the differences between age groups (multigroup SEM) or between people with different ages (multidimensional SEM). Without taking into account item bias older people would have been estimated to be less depressed than younger people, whereas after taking into account item bias this difference was no longer significant. The detected item biases indicated that younger people experience more depression symptoms as compared to older people, relative to the level of depression. In contrast, the gender- and age-related biases that were detected in the items of the anxiety subscale of the HADS did not lead to different conclusions at the subscale level. A possible explanation for these results is that the detected item biases canceled each other out at the subscale level. In general, the investigation of item bias is important for a valid comparison of scores both at the item level and at the subscale level. Moreover, indications of item bias may improve our understanding of (possible) differences between groups of patients. The results from the present study support valid comparisons between men and women on both the anxiety and depression subscales of the HADS, whereas a valid comparison between people of different ages is only supported for the anxiety subscale, but not for the depression subscale. Of course, depression in people of different ages can still be validly compared whether DIF is taken into account, for example by allowing for partial invariance of item parameters in multigroup SEM, or by allowing for a number of direct age effects in multidimensional SEM.

In the present paper, nonuniform bias was only investigated with the multigroup SEM approach but not with the multidimensional SEM approach. Although it has been shown that investigation of nonuniform item bias is possible by including interaction terms between the underlying trait of interest and the other exogenous variables [3], these types of extensions are not easily implemented and were therefore not applied. Even though possible nonuniform item bias thus remained undetected within the multidimensional SEM approach, the results of uniform bias detection were largely consistent between both SEM approaches. Both SEM approaches identified the same items with uniform gender- and/or age-related bias in the anxiety subscale of the HADS. In addition, the detection of uniform age-related bias in the depression subscale of the HADS was largely consistent across SEM approaches (with agreement on four items), although less so with regard to the detection of gender-related bias.

Differences between the two SEM approaches in terms of the detection of uniform item bias can occur because of several reasons. First, multidimensional SEM takes the relation between anxiety and depression into account during the bias detection procedure and multigroup SEM does not. Second, when age and gender are related, then age-related item bias may be detected in the multigroup SEM approach only because there exists gender-related item bias (or vice versa), whereas in the multidimensional SEM approach possible relations between gender and age are taken into account. In the present application, some of the age-related item bias may have sufficiently explained some of the gender differences on these items that were only detected by the multigroup SEM approach. In addition, gender biases that were in the opposite direction of the age-related bias found in the same items might have been obscured in the multigroup SEM approach due to the association between gender and age, although the correlation in our empirical example was only small. Finally, the multidimensional SEM approach may have larger power to detect uniform item bias, as it is based on the entire sample rather than subsamples.

The results of Cameron et al. [7] were consistent with the results of uniform bias detection from both SEM approaches applied in the present paper, as the SEM methods generally identified the same items as biased. However, the SEM approaches did identify more items with uniform bias. We cannot know whether the methods have correctly identified items with bias, and/or whether some biased items have been missed. Nevertheless, consistency in the identification of bias across different detection methods may give some confidence in the robustness of results. The detected uniform item biases in the anxiety subscale of the HADS were largely equivalent across the different bias detection methods, and could be substantively interpreted. However, the pattern of detected uniform item biases in the depression subscale of the HADS was less consistent across the different detection methods and both SEM methods identified more items with uniform bias. It could be that SEM has more power to detect effects, but we cannot exclude the possibility of false detection. To further investigate and compare the appropriateness of the different item bias detection methods, simulation studies would be required to investigate whether uniform and nonuniform item bias can be correctly identified. In such a simulation study one could, for example, investigate the performance of these different approaches under different circumstances, e.g., the size of the item bias, the direction of the item bias, the type of item bias, the number of items affected by bias.

To conclude, both the multigroup SEM approach and multidimensional SEM approach can be applied to detect bias in observed item scores. Advantages of the multigroup SEM approach are that it uses a latent trait operationalization, it can detect both uniform and nonuniform bias, and possible item bias can be taken into account to assess true differences between groups. In addition, the extension to multidimensional models enables the investigation of item bias with respect to any factor or variable (e.g., continuous or categorical, latent or manifest), where continuous variables can be included in the model without categorizing them, and item bias can be investigated with respect to several variables simultaneously. Although detection of nonuniform bias with multidimensional SEM is less straightforward, it can be implemented and has been shown to perform well [3]. Therefore, the SEM method provides a flexible tool for the investigation of item bias in health-related questionnaires and may thus ultimately provide a more valid comparison of patients’ well-being that is relevant for both research and clinical practice.

## Electronic supplementary material

Below is the link to the electronic supplementary material.

Supplementary material 1 (DOCX 22 kb)

Supplementary material 2 (DOCX 38 kb)
