# Supplementary material for: Item bias detection in the Hospital Anxiety and Depression Scale using structural equation modeling: comparison with other item bias detection methods
Source: Qual Life Res. 2016 Dec 9;26(6):1439–50. doi: 10.1007/s11136-016-1469-1 (PMC5420371; doi:10.1007/s11136-016-1469-1)
Supplement: Supplementary file 1 — Supplementary material 1 (DOCX 22 kb) [file 11136_2016_1469_MOESM1_ESM.docx]

APPENDIX A

STAGE 1: PRELIS SYNTAXES FOR ANALYSES OF ALL ITEMS OF THE HADS

- MULTIGROUP SEM APPROACH P.2

- MULTIDIMENSIONAL SEM APPROACH P.7

###########################################################################

# MULTIGROUP SEM APPROACH

###########################################################################

STEP 1: BIVARIATE NORMALITY

Data Ninputvariables = 17

Labels

A1R D1 A2R D2 A3R D3R A4 D4R A5 D5R A6R D6 A7R D7 Sex Age Age_cat

Rawdata=HADS.RAW RE

CLabels Sex 1=male 2=female

CLabels Age_cat 0=young 1=old

! women

Sdelete Sex=2

Select A1R - D7

Output MA=PM

Data Ninputvariables = 17

Labels

A1R D1 A2R D2 A3R D3R A4 D4R A5 D5R A6R D6 A7R D7 Sex Age Age_cat

Rawdata=HADS.RAW RE

CLabels Sex 1=male 2=female

CLabels Age_cat 0=young 1=old

! men

Sdelete Sex=1

Select A1R - D7

Output MA=PM

Data Ninputvariables = 17

Labels

A1R D1 A2R D2 A3R D3R A4 D4R A5 D5R A6R D6 A7R D7 Sex Age Age_cat

Rawdata=HADS.RAW RE

CLabels Sex 1=male 2=female

CLabels Age_cat 0=young 1=old

! Age_cat < 65

Sdelete Age_cat=0

Select A1R - D7

Output MA=PM

Data Ninputvariables = 17

Labels

A1R D1 A2R D2 A3R D3R A4 D4R A5 D5R A6R D6 A7R D7 Sex Age Age_cat

Rawdata=HADS.RAW RE

CLabels Sex 1=male 2=female

CLabels Age_cat 0=young 1=old

! Age_cat > 65

Sdelete Age_cat=1

Select A1R - D7

Output MA=PM

STEP 2: INVARIANT THRESHOLDS

Data Ninputvariables = 17

Labels

A1R D1 A2R D2 A3R D3R A4 D4R A5 D5R A6R D6 A7R D7 Sex Age Age_cat

Rawdata=HADS.RAW RE

CLabels Sex 1=male 2=female

CLabels Age_cat 0=young 1=old

Select A1R - D7

Output MA=PM TH=HADS.TH

Data Ninputvariables = 17

Labels

A1R D1 A2R D2 A3R D3R A4 D4R A5 D5R A6R D6 A7R D7 Sex Age Age_cat

Rawdata=HADS.RAW RE

CLabels Sex 1=male 2=female

CLabels Age_cat 0=young 1=old

! women

Sdelete Sex=2

Select A1R - D7

FT=HADS.TH A1R

FT D1

FT A2R

FT D2

FT A3R

FT D3R

FT A4

FT D4R

FT A5

FT D5R

FT A6R

FT D6

FT A7R

FT D7

Output MA=PM

Data Ninputvariables = 17

Labels

A1R D1 A2R D2 A3R D3R A4 D4R A5 D5R A6R D6 A7R D7 Sex Age Age_cat

Rawdata=HADS.RAW RE

CLabels Sex 1=male 2=female

CLabels Age_cat 0=young 1=old

! men

Sdelete Sex=1

Select A1R - D7

FT=HADS.TH A1R

FT D1

FT A2R

FT D2

FT A3R

FT D3R

FT A4

FT D4R

FT A5

FT D5R

FT A6R

FT D6

FT A7R

FT D7

Output MA=PM

Data Ninputvariables = 17

Labels

A1R D1 A2R D2 A3R D3R A4 D4R A5 D5R A6R D6 A7R D7

Sex Age Age_cat

Rawdata=HADS.RAW RE

CLabels Sex 1=male 2=female

CLabels Age_cat 0=young 1=old

! age < 65

Sdelete Age_cat=0

Select A1R – D7

FT=HADS.TH A1R

FT D1

FT A2R

FT D2

FT A3R

FT D3R

FT A4

FT D4R

FT A5

FT D5R

FT A6R

FT D6

FT A7R

FT D7

Output MA=PM

Data Ninputvariables = 17

Labels

A1R D1 A2R D2 A3R D3R A4 D4R A5 D5R A6R D6 A7R D7

Sex Age Age_cat

Rawdata=HADS.RAW RE

CLabels Sex 1=male 2=female

CLabels Age_cat 0=young 1=old

! Age_cat > 65

Sdelete Age_cat=1

Select A1R – D7

FT=HADS.TH A1R

FT D1

FT A2R

FT D2

FT A3R

FT D3R

FT A4

FT D4R

FT A5

FT D5R

FT A6R

FT D6

FT A7R

FT D7

Output MA=PM

STEP 3: COMPUTE POLYCHORIC CORRELATIONS, VARIANCES AND MEAN VECTOR

Data Ninputvariables = 17

Labels

A1R D1 A2R D2 A3R D3R A4 D4R A5 D5R A6R D6 A7R D7

Sex Age Age_cat

Rawdata=HADS.RAW RE

CLabels Sex 1=male 2=female

CLabels Age_cat 0=young 1=old

! women

Sdelete Sex=2

Select A1R - D7

FT=HADS.TH A1R

FT D1

FT A2R

FT D2

FT A3R

FT D3R

FT A4

FT D4R

FT A5

FT D5R

FT A6R

FT D6

FT A7R

FT D7

Output MA=CM CM=HADS_F.CM AC=HADS_F.ACC ME=HADS_F.ME

Data Ninputvariables = 17

Labels

A1R D1 A2R D2 A3R D3R A4 D4R A5 D5R A6R D6 A7R D7

Sex Age Age_cat

Rawdata=HADS.RAW RE

CLabels Sex 1=male 2=female

CLabels Age_cat 0=young 1=old

! men

Sdelete Sex=1

Select A1R - D7

FT=HADS.TH A1R

FT D1

FT A2R

FT D2

FT A3R

FT D3R

FT A4

FT D4R

FT A5

FT D5R

FT A6R

FT D6

FT A7R

FT D7

Output MA=CM CM=HADS_M.CM AC=HADS_M.ACC ME=HADS_M.ME

Data Ninputvariables = 17

Labels

A1R D1 A2R D2 A3R D3R A4 D4R A5 D5R A6R D6 A7R D7

Sex Age Age_cat

Rawdata=HADS.RAW RE

CLabels Sex 1=male 2=female

CLabels Age_cat 0=young 1=old

! age < 65

Sdelete Age_cat=0

Select A1R – D7

FT=HADS.TH A1R

FT D1

FT A2R

FT D2

FT A3R

FT D3R

FT A4

FT D4R

FT A5

FT D5R

FT A6R

FT D6

FT A7R

FT D7

Output MA=CM CM=HADS_Y.CM AC=HADS_Y.ACC ME=HADS_Y.ME

Data Ninputvariables = 17

Labels

A1R D1 A2R D2 A3R D3R A4 D4R A5 D5R A6R D6 A7R D7

Sex Age Age_cat

Rawdata=HADS.RAW RE

CLabels Sex 1=male 2=female

CLabels Age_cat 0=young 1=old

! Age_cat > 65

Sdelete Age_cat=1

Select A1R – D7

FT=HADS.TH A1R

FT D1

FT A2R

FT D2

FT A3R

FT D3R

FT A4

FT D4R

FT A5

FT D5R

FT A6R

FT D6

FT A7R

FT D7

Output MA=CM CM=HADS_O.CM AC=HADS_O.ACC ME=HADS_O.ME

###########################################################################

# MULTIDIMENSIONAL SEM APPROACH

###########################################################################

STEP 1: BIVARIATE NORMALITY

Data Ninputvariables = 17

Labels

A1R D1 A2R D2 A3R D3R A4 D4R A5 D5R A6R D6 A7R D7 Sex Age Age_cat

Rawdata=HADS.RAW RE

CLabels Sex 1=male 2=female

CLabels Age_cat 0=young 1=old

Select A1R – Age

Output MA=PM

STEP 2: COMPUTE POLYCHORIC CORRELATIONS, VARIANCES AND MEAN VECTOR

Data Ninputvariables = 17

Labels

A1R D1 A2R D2 A3R D3R A4 D4R A5 D5R A6R D6 A7R D7 Sex Age Age_cat

Rawdata=HADS.RAW RE

CLabels Sex 1=male 2=female

CLabels Age_cat 0=young 1=old

Select A1R – Age

Output MA=CM CM=HADSall.CM AC=HADSall.ACC ME=HADSall.ME
