# Supplementary material for: Item bias detection in the Hospital Anxiety and Depression Scale using structural equation modeling: comparison with other item bias detection methods
Source: Qual Life Res. 2016 Dec 9;26(6):1439–50. doi: 10.1007/s11136-016-1469-1 (PMC5420371; doi:10.1007/s11136-016-1469-1)
Supplement: Supplementary file 2 — Supplementary material 2 (DOCX 38 kb) [file 11136_2016_1469_MOESM2_ESM.docx]

APPENDIX B

LISREL SYNTAXES FOR ANALYSES OF GENDER- AND AGE-RELATED ITEM BIAS IN THE HOSPITAL ANXIETY AND DEPRESSION SCALE

- MULTIGROUP SEM APPROACH: GENDER-RELATED ITEM BIAS HADS-A P.2

- MULTIGROUP SEM APPROACH: AGE-RELATED ITEM BIAS HADS-A P.7

- MULTIGROUP SEM APPROACH: GENDER-RELATED ITEM BIAS HADS-D P.12

- MULTIGROUP SEM APPROACH: AGE-RELATED ITEM BIAS HADS-D P.17

- MULTIDIMENSIONAL SEM APPROACH: GENDER- AND AGE-RELATED ITEM BIAS P.22

IN THE HADS-A AND HADS-D

###########################################################################

# MULTIGROUP SEM APPROACH: GENDER-RELATED ITEM BIAS HADS-A

###########################################################################

STEP 1 : MEASUREMENT MODEL

! female

da ng=2 ni=14 no=633

cm fi=HADS_F.CM RE

me fi=HADS_F.ME RE

wm = HADS_F.ACC RE

LA

A1R D1 A2R D2 A3R D3R A4 D4R A5 D5R A6R D6 A7R D7

SE

A1R A2R A3R A4 A5 A6R A7R /

mo ny=7 ne=1 ly=fu,fr ps=sy,fi te=sy,fr al=fu,fi ty=fu,fr

LE

ANX

ma ps

1

pa ly

1

1

1

1

1

1

1

pa te

1

0 1

0 0 1

0 0 0 1

0 0 0 0 1

0 0 0 0 0 1

0 0 0 0 0 0 1

ou dwls rs so mi

! male

title male

da ni=14 no=435

cm fi=HADS_M.CM RE

me fi=HADS_M.ME RE

wm = HADS_M.ACC RE

LA

A1R D1 A2R D2 A3R D3R A4 D4R A5 D5R A6R D6 A7R D7

SE

A1R A2R A3R A4 A5 A6R A7R /

mo ny=7 ne=1 ly=fu,fr ps=sy,fi te=sy,fr al=fu,fi ty=fu,fr

LE

ANX

ma ps

1

pa ly

1

1

1

1

1

1

1

pa te

1

0 1

0 0 1

0 0 0 1

0 0 0 0 1

0 0 0 0 0 1

0 0 0 0 0 0 1

pa ty

9 10 11 12 13 14 15

pd

ou dwls rs so

STEP 2 : NO ITEM BIAS MODEL

! female

da ng=2 ni=14 no=633

cm fi=HADS_F.CM RE

me fi=HADS_F.ME RE

wm = HADS_F.ACC RE

LA

A1R D1 A2R D2 A3R D3R A4 D4R A5 D5R A6R D6 A7R D7

SE

A1R A2R A3R A4 A5 A6R A7R /

mo ny=7 ne=1 ly=fu,fr ps=sy,fi te=sy,fr al=fu,fi ty=fu,fr

LE

ANX

ma ps

1

pa ly

2

3

4

5

6

7

8

pa te

1

0 1

0 0 1

0 0 0 1

0 0 0 0 1

0 0 0 0 0 1

0 0 0 0 0 0 1

pa ty

9 10 11 12 13 14 15

ou dwls rs so

! male

title male

da ni=14 no=435

cm fi=HADS_M.CM RE

me fi=HADS_M.ME RE

wm = HADS_M.ACC RE

LA

A1R D1 A2R D2 A3R D3R A4 D4R A5 D5R A6R D6 A7R D7

SE

A1R A2R A3R A4 A5 A6R A7R /

mo ny=7 ne=1 ly=fu,fr ps=sy,fr te=sy,fr al=fu,fr ty=fu,fr

LE

ANX

ma ps

1

pa ly

2

3

4

5

6

7

8

pa te

1

0 1

0 0 1

0 0 0 1

0 0 0 0 1

0 0 0 0 0 1

0 0 0 0 0 0 1

pa ty

9 10 11 12 13 14 15

ou dwls rs so mi

STEP 3: FINAL MODEL

! female

da ng=2 ni=14 no=633

cm fi=HADS_F.CM RE

me fi=HADS_F.ME RE

wm=HADS_F.ACC RE

LA

A1R D1 A2R D2 A3R D3R A4 D4R A5 D5R A6R D6 A7R D7

SE

A1R A2R A3R A4 A5 A6R A7R /

mo ny=7 ne=1 ly=fu,fr ps=sy,fi te=sy,fr al=fu,fi ty=fu,fr

LE

ANX

ma ps

1

pa ly

2

3

4

5

6

7

8

pa te

1

0 1

0 0 1

0 0 0 1

0 0 0 0 1

0 0 0 0 0 1

0 0 0 0 0 0 1

pa ty

9 10 11 12 **213 214** 15 ! TWO UNIFORM ITEM BIASES

ou dwls rs so

! male

title male

da ni=14 no=435

cm fi=HADS_M.CM RE

me fi=HADS_M.ME RE

wm=HADS_M.ACC RE

LA

A1R D1 A2R D2 A3R D3R A4 D4R A5 D5R A6R D6 A7R D7

SE

A1R A2R A3R A4 A5 A6R A7R /

mo ny=7 ne=1 ly=fu,fr ps=sy,fr te=sy,fr al=fu,fr ty=fu,fr

LE

ANX

ma ps

1

pa ly

2

3

4

5

6

7

8

pa te

1

0 1

0 0 1

0 0 0 1

0 0 0 0 1

0 0 0 0 0 1

0 0 0 0 0 0 1

pa ty

9 10 11 12 13 14 15

ou dwls rs so mi

###########################################################################

# MULTIGROUP SEM APPROACH: AGE-RELATED ITEM BIAS HADS-A

###########################################################################

STEP 1: MEASUREMENT MODEL

! age group < 65 years

da ng=2 ni=14 no=814

cm fi=HADS_Y.CM RE

me fi=HADS_y.ME RE

wm = HADS_Y.ACC RE

LA

A1R D1 A2R D2 A3R D3R A4 D4R A5 D5R A6R D6 A7R D7

SE

A1R A2R A3R A4 A5 A6R A7R /

mo ny=7 ne=1 ly=fu,fr ps=sy,fi te=sy,fr al=fu,fi ty=fu,fr

LE

ANX

ma ps

1

pa ly

1

1

1

1

1

1

1

pa te

1

0 1

0 0 1

0 0 0 1

0 0 0 0 1

0 0 0 0 0 1

0 0 0 0 0 0 1

ou dwls rs so mi

! age group > 65 years

da ni=14 no=254

cm fi=HADS_O.CM RE

me fi=HADS_O.ME RE

wm=HADS_O.ACC RE

LA

A1R D1 A2R D2 A3R D3R A4 D4R A5 D5R A6R D6 A7R D7

SE

A1R A2R A3R A4 A5 A6R A7R /

mo ny=7 ne=1 ly=fu,fr ps=sy,fi te=sy,fr al=fu,fi ty=fu,fr

LE

ANX

ma ps

1

pa ly

1

1

1

1

1

1

1

pa te

1

0 1

0 0 1

0 0 0 1

0 0 0 0 1

0 0 0 0 0 1

0 0 0 0 0 0 1

pd

ou dwls rs so mi

STEP 2: NO ITEM BIAS MODEL

! age group < 65 years

da ng=2 ni=14 no=814

cm fi=HADS_Y.CM RE

me fi=HADS_Y.ME RE

wm=HADS_Y.ACC RE

LA

A1R D1 A2R D2 A3R D3R A4 D4R A5 D5R A6R D6 A7R D7

SE

A1R A2R A3R A4 A5 A6R A7R /

mo ny=7 ne=1 ly=fu,fr ps=sy,fi te=sy,fr al=fu,fi ty=fu,fr

LE

ANX

ma ps

1

pa ly

2

3

4

5

6

7

8

pa te

1

0 1

0 0 1

0 0 0 1

0 0 0 0 1

0 0 0 0 0 1

0 0 0 0 0 0 1

pa ty

9 10 11 12 13 14 15

ou dwls rs so

! age group > 65 years

da ni=14 no=254

cm fi=HADS_O.CM RE

me fi=HADS_O.ME RE

wm=HADS_O.ACC RE

LA

A1R D1 A2R D2 A3R D3R A4 D4R A5 D5R A6R D6 A7R D7

SE

A1R A2R A3R A4 A5 A6R A7R /

mo ny=7 ne=1 ly=fu,fr ps=sy,fr te=sy,fr al=fu,fr ty=fu,fr

LE

ANX

ma ps

1

pa ly

2

3

4

5

6

7

8

pa te

1

0 1

0 0 1

0 0 0 1

0 0 0 0 1

0 0 0 0 0 1

0 0 0 0 0 0 1

pa ty

9 10 11 12 13 14 15

ou dwls rs so mi

STEP 3: FINAL MODEL

! age group < 65 years

da ng=2 ni=14 no=814

cm fi=HADS_Y.CM RE

me fi=HADS_Y.ME RE

wm = HADS_Y.ACC RE

LA

A1R D1 A2R D2 A3R D3R A4 D4R A5 D5R A6R D6 A7R D7

SE

A1R A2R A3R A4 A5 A6R A7R /

mo ny=7 ne=1 ly=fu,fr ps=sy,fi te=sy,fr al=fu,fi ty=fu,fr

LE

ANX

ma ps

1

pa ly

2

**33** ! NONUNIFORM ITEM BIAS

4

5

6

7

8

pa te

1

0 1

0 0 1

0 0 0 1

0 0 0 0 1

0 0 0 0 0 1

0 0 0 0 0 0 1

pa ty

**29** 10 11 12 13 14 **215** ! TWO UNIFORM ITEM BIASES

ou dwls rs so

! age group > 65 years

da ni=14 no=254

cm fi=HADS_O.CM RE

me fi=HADS_O.ME RE

wm=HADS_O.ACC RE

LA

A1R D1 A2R D2 A3R D3R A4 D4R A5 D5R A6R D6 A7R D7

SE

A1R A2R A3R A4 A5 A6R A7R /

mo ny=7 ne=1 ly=fu,fr ps=sy,fr te=sy,fr al=fu,fr ty=fu,fr

LE

ANX

ma ps

1

pa ly

2

3

4

5

6

7

8

pa te

1

0 1

0 0 1

0 0 0 1

0 0 0 0 1

0 0 0 0 0 1

0 0 0 0 0 0 1

pa ty

9 10 11 12 13 14 15

ou dwls rs so mi

###########################################################################

# MULTIGROUP SEM APPROACH: GENDER-RELATED ITEM BIAS HADS-D

###########################################################################

STEP 1: MEASUREMENT MODEL

! female

da ng=2 ni=14 no=633

cm fi=HADS_F.CM RE

me fi=HADS_F.ME RE

wm = HADS_F.ACC RE

LA

A1R D1 A2R D2 A3R D3R A4 D4R A5 D5R A6R D6 A7R D7

SE

D1 D2 D3R D4R D5R D6 D7 /

mo ny=7 ne=1 ly=fu,fr ps=sy,fi te=sy,fr al=fu,fi ty=fu,fr

LE

DEP

ma ps

1

pa ly

1

1

1

1

1

1

1

pa te

1

0 1

0 0 1

0 0 0 1

0 0 0 0 1

0 0 0 0 0 1

0 0 0 0 0 0 1

ou dwls rs so mi

! male

title male

da ni=14 no=435

cm fi=HADS_M.CM RE

me fi=HADS_M.ME RE

wm=HADS_M.ACC RE

LA

A1R D1 A2R D2 A3R D3R A4 D4R A5 D5R A6R D6 A7R D7

SE

D1 D2 D3R D4R D5R D6 D7 /

mo ny=7 ne=1 ly=fu,fr ps=sy,fi te=sy,fr al=fu,fi ty=fu,fr

LE

DEP

ma ps

1

pa ly

1

1

1

1

1

1

1

pa te

1

0 1

0 0 1

0 0 0 1

0 0 0 0 1

0 0 0 0 0 1

0 0 0 0 0 0 1

pd

ou dwls rs so mi

STEP 2: NO ITEM BIAS MODEL

! female

da ng=2 ni=14 no=633

cm fi=HADS_F.CM RE

me fi=HADS_F.ME RE

wm=HADS_F.ACC RE

LA

A1R D1 A2R D2 A3R D3R A4 D4R A5 D5R A6R D6 A7R D7

SE

D1 D2 D3R D4R D5R D6 D7 /

mo ny=7 ne=1 ly=fu,fr ps=sy,fi te=sy,fr al=fu,fi ty=fu,fr

LE

DEP

ma ps

1

pa ly

2

3

4

5

6

7

8

pa te

1

0 1

0 0 1

0 0 0 1

0 0 0 0 1

0 0 0 0 0 1

0 0 0 0 0 0 1

pa ty

9 10 11 12 13 14 15

ou dwls rs so mi

! male

title male

da ni=14 no=435

cm fi=HADS_M.CM RE

me fi=HADS_M.ME RE

wm=HADS_M.ACC RE

LA

A1R D1 A2R D2 A3R D3R A4 D4R A5 D5R A6R D6 A7R D7

SE

D1 D2 D3R D4R D5R D6 D7 /

mo ny=7 ne=1 ly=fu,fr ps=sy,fr te=sy,fr al=fu,fr ty=fu,fr

LE

DEP

ma ps

1

pa ly

2

3

4

5

6

7

8

pa te

1

0 1

0 0 1

0 0 0 1

0 0 0 0 1

0 0 0 0 0 1

0 0 0 0 0 0 1

pa ty

9 10 11 12 13 14 15

ou dwls rs so mi

STEP 3: FINAL MODEL

! female

da ng=2 ni=14 no=633

cm fi=HADS_F.CM RE

me fi=HADS_F.ME RE

wm=HADS_F.ACC RE

LA

A1R D1 A2R D2 A3R D3R A4 D4R A5 D5R A6R D6 A7R D7

SE

D1 D2 D3R D4R D5R D6 D7 /

mo ny=7 ne=1 ly=fu,fr ps=sy,fi te=sy,fr al=fu,fi ty=fu,fr

LE

DEP

ma ps

1

pa ly

2

**23** ! NONUNIFORM ITEM BIAS

4

5

**26** ! NONUNIFORM ITEM BIAS

7

8

pa te

1

0 1

0 0 1

0 0 0 1

0 0 0 0 1

0 0 0 0 0 1

0 0 0 0 0 0 1

pa ty

9 **210** 11 12 13 14 15 ! ONE UNIFORM ITEM BIAS

ou dwls rs so mi

! male

title male

da ni=14 no=435

cm fi=HADS_M.CM RE

me fi=HADS_M.ME RE

wm=HADS_M.ACC RE

LA

A1R D1 A2R D2 A3R D3R A4 D4R A5 D5R A6R D6 A7R D7

SE

D1 D2 D3R D4R D5R D6 D7 /

mo ny=7 ne=1 ly=fu,fr ps=sy,fr te=sy,fr al=fu,fr ty=fu,fr

LE

DEP

ma ps

1

pa ly

2

3

4

5

6

7

8

pa te

1

0 1

0 0 1

0 0 0 1

0 0 0 0 1

0 0 0 0 0 1

0 0 0 0 0 0 1

pa ty

9 10 11 12 13 14 15

ou dwls rs so mi

###########################################################################

# MULTIGROUP SEM APPROACH: AGE-RELATED ITEM BIAS HADS-D ###########################################################################

STEP 1: MEASUREMENT MODEL

! age group < 65

da ng=2 ni=14 no=814

cm fi=HADS_Y.CM RE

me fi=HADS_Y.ME RE

wm = HADS_Y.ACC RE

LA

A1R D1 A2R D2 A3R D3R A4 D4R A5 D5R A6R D6 A7R D7

SE

D1 D2 D3R D4R D5R D6 D7 /

mo ny=7 ne=1 ly=fu,fr ps=sy,fi te=sy,fr al=fu,fi ty=fu,fr

LE

DEP

ma ps

1

pa ly

1

1

1

1

1

1

1

pa te

1

0 1

0 0 1

0 0 0 1

0 0 0 0 1

0 0 0 0 0 1

0 0 0 0 0 0 1

pa ty

1 1 1 1 1 1 1

ou dwls rs so

! age group > 65

da ni=14 no=254

cm fi=HADS_O.CM RE

me fi=HADS_O.ME RE

wm=HADS_O.ACC RE

LA

A1R D1 A2R D2 A3R D3R A4 D4R A5 D5R A6R D6 A7R D7

SE

D1 D2 D3R D4R D5R D6 D7 /

mo ny=7 ne=1 ly=fu,fr ps=sy,fi te=sy,fr al=fu,fi ty=fu,fr

LE

DEP

ma ps

1

pa ly

1

1

1

1

1

1

1

pa te

1

0 1

0 0 1

0 0 0 1

0 0 0 0 1

0 0 0 0 0 1

0 0 0 0 0 0 1

pa ty

1 1 1 1 1 1 1

ou dwls rs so

STEP 2: NO ITEM BIAS MODEL

! age group < 65

da ng=2 ni=14 no=814

cm fi=HADS_Y.CM RE

me fi=HADS_Y.ME RE

wm = HADS_Y.ACC RE

LA

A1R D1 A2R D2 A3R D3R A4 D4R A5 D5R A6R D6 A7R D7

SE

D1 D2 D3R D4R D5R D6 D7 /

mo ny=7 ne=1 ly=fu,fr ps=sy,fi te=sy,fr al=fu,fi ty=fu,fr

LE

DEP

ma ps

1

pa ly

2

3

4

5

6

7

8

pa te

1

0 1

0 0 1

0 0 0 1

0 0 0 0 1

0 0 0 0 0 1

0 0 0 0 0 0 1

pa ty

9 10 11 12 13 14 15

ou dwls rs so

! age group > 65

da ni=14 no=254

cm fi=HADS_O.CM RE

me fi=HADS_O.ME RE

wm = HADS_O.ACC RE

LA

A1R D1 A2R D2 A3R D3R A4 D4R A5 D5R A6R D6 A7R D7

SE

D1 D2 D3R D4R D5R D6 D7 /

mo ny=7 ne=1 ly=fu,fr ps=sy,fr te=sy,fr al=fu,fr ty=fu,fr

LE

DEP

ma ps

1

pa ly

2

3

4

5

6

7

8

pa te

1

0 1

0 0 1

0 0 0 1

0 0 0 0 1

0 0 0 0 0 1

0 0 0 0 0 0 1

pa ty

9 10 11 12 13 14 15

ou dwls rs so mi

STEP 3: FINAL MODEL

! age group < 65

da ng=2 ni=14 no=814

cm fi=HADS_Y.CM RE

me fi=HADS_Y.ME RE

wm = HADS_Y.ACC RE

LA

A1R D1 A2R D2 A3R D3R A4 D4R A5 D5R A6R D6 A7R D7

SE

D1 D2 D3R D4R D5R D6 D7 /

mo ny=7 ne=1 ly=fu,fr ps=sy,fi te=sy,fr al=fu,fi ty=fu,fr

LE

DEP

ma ps

1

pa ly

2

**23** ! NONUNIFORM ITEM BIAS

4

5

6

**27** ! NONUNIFORM ITEM BIAS

8

pa te

1

0 1

0 0 1

0 0 0 1

0 0 0 0 1

0 0 0 0 0 1

0 0 0 0 0 0 1

pa ty

9 **210 211** 12 **213** 14 **215** ! FOUR UNIFORM ITEM BIASES

ou dwls rs so

! age group > 65

da ni=14 no=254

cm fi=HADS_O.CM RE

me fi=HADS_O.ME RE

wm = HADS_O.ACC RE

LA

A1R D1 A2R D2 A3R D3R A4 D4R A5 D5R A6R D6 A7R D7

SE

D1 D2 D3R D4R D5R D6 D7 /

mo ny=7 ne=1 ly=fu,fr ps=sy,fr te=sy,fr al=fu,fr ty=fu,fr

LE

DEP

ma ps

1

pa ly

2

3

4

5

6

7

8

pa te

1

0 1

0 0 1

0 0 0 1

0 0 0 0 1

0 0 0 0 0 1

0 0 0 0 0 0 1

pa ty

9 10 11 12 13 14 15

ou dwls rs so mi

###########################################################################

# MULTIDIMENSIONAL SEM APPROACH: GENDER- AND AGE-RELATED ITEM BIAS IN THE

# HADS-A AND HADS-D

###########################################################################

STEP 1: MEASUREMENT MODEL

! all data

da ng=1 ni=16 no=1068 ma=cm

cm fi=HADSall.CM RE

me fi=HADSall.ME RE

wm = HADSall.ACC RE

LA

A1R D1 A2R D2 A3R D3R A4 D4R A5 D5R A6R D6 A7R D7 Sex Age

SE

A1R A2R A3R A4 A5 A6R A7R D1 D2 D3R D4R D5R D6 D7 /

mo ny=14 ne=2 ly=fu,fr ps=sy,fi te=sy,fr al=fu,fi ty=fu,fr

LE

ANX DEP SEX AGE

ma ps

1

0 1

pa ps

0

1 0

pa ly

1 0

1 0

1 0

1 0

1 0

1 0

1 0

0 1

0 1

0 1

0 1

0 1

0 1

0 1

pa te

1

0 1

0 0 1

0 0 0 1

0 0 0 0 1

0 0 0 0 0 1

0 0 0 0 0 0 1

0 0 0 0 0 0 0 1

0 0 0 0 0 0 0 0 1

0 0 0 0 0 0 0 0 0 1

0 0 0 0 0 0 0 0 0 0 1

0 0 0 0 0 0 0 0 0 0 0 1

0 0 0 0 0 0 0 0 0 0 0 0 1

0 0 0 0 0 0 0 0 0 0 0 0 0 1

ou dwls so sc mi

STEP 2: NO ITEM BIAS MODEL

! all data

da ng=1 ni=16 no=1068 ma=cm

cm fi=HADSall.CM RE

me fi=HADSall.ME RE

wm=HADSall.ACC RE

LA

A1R D1 A2R D2 A3R D3R A4 D4R A5 D5R A6R D6 A7R D7 Sex Age

SE

A1R A2R A3R A4 A5 A6R A7R D1 D2 D3R D4R D5R D6 D7 Sex Age /

mo ny=16 ne=4 ly=fu,fr ps=sy,fi te=sy,fr al=fu,fi ty=fu,fr

LE

ANX DEP SEX AGE

ma ps

1

0 1

0 0 0

0 0 0 0

pa ps

0

1 0

1 1 1

1 1 1 1

ma al

0 0 0 0

pa al

0 0 1 1

fi ty 15 ty 16

va 0 ty 15 ty 16

pa ly

1 0 0 0

1 0 0 0

1 0 0 0

1 0 0 0

1 0 0 0

1 0 0 0

1 0 0 0

0 1 0 0

0 1 0 0

0 1 0 0

0 1 0 0

0 1 0 0

0 1 0 0

0 1 0 0

0 0 0 0

0 0 0 0

fi ly 15 3 ly 16 4

va 1 ly 15 3 ly 16 4

pa te

1

0 1

0 0 1

0 0 0 1

0 0 0 0 1

0 0 0 0 0 1

0 0 0 0 0 0 1

0 0 0 0 0 0 0 1

0 0 0 0 0 0 0 0 1

0 0 0 0 0 0 0 0 0 1

0 0 0 0 0 0 0 0 0 0 1

0 0 0 0 0 0 0 0 0 0 0 1

0 0 0 0 0 0 0 0 0 0 0 0 1

0 0 0 0 0 0 0 0 0 0 0 0 0 1

0 0 0 0 0 0 0 0 0 0 0 0 0 0 1

0 0 0 0 0 0 0 0 0 0 0 0 0 0 0 1

fi te 15 15 te 16 16

va 0 te 15 15 te 16 16

ou dwls so sc

STEP 3: FINAL MODEL

! all data

da ng=1 ni=16 no=1068 ma=cm

cm fi=HADSall.CM RE

me fi=HADSall.ME RE

wm = HADSall.ACC RE

LA

A1R D1 A2R D2 A3R D3R A4 D4R A5 D5R A6R D6 A7R D7 Sex Age

SE

A1R A2R A3R A4 A5 A6R A7R D1 D2 D3R D4R D5R D6 D7 Sex Age /

mo ny=16 ne=4 ly=fu,fr ps=sy,fi te=sy,fr al=fu,fi ty=fu,fr

LE

ANX DEP SEX AGE

ma ps

1

0 1

0 0 0

0 0 0 0

pa ps

0

1 0

1 1 1

1 1 1 1

ma al

0 0 0 0

pa al

0 0 1 1

fi ty 15 ty 16

va 0 ty 15 ty 16

pa ly

1 0 0 **1** ! UNIFORM AGE-RELATED BIAS HADS-A

1 0 0 0

1 0 0 0

1 0 0 0

1 0 **1** 0 ! UNIFORM GENDER-RELATED BIAS HADS-A

1 0 **1** 0 ! UNIFORM GENDER-RELATED BIAS HADS-A

1 0 0 **1** ! UNIFORM AGE-RELATED BIAS HADS-A

0 1 **1** 0 ! UNIFORM GENDER-RELATED BIAS HADS-D

0 1 0 **1** ! UNIFORM AGE-RELATED BIAS HADS-D

0 1 **1 1** ! UNIFORM GENDER-RELATED BIAS HADS-D

0 1 0 **1** ! UNIFORM AGE-RELATED BIAS HADS-D

0 1 0 **1** ! UNIFORM AGE-RELATED BIAS HADS-D

0 1 0 0

0 1 **1 1** ! UNIFORM GENDER- AND AGE-RELATED BIAS HADS-D

0 0 0 0

0 0 0 0

fi ly 15 3 ly 16 4

va 1 ly 15 3 ly 16 4

pa te

1

0 1

0 0 1

0 0 0 1

0 0 0 0 1

0 0 0 0 0 1

0 0 0 0 0 0 1

0 0 0 0 0 0 0 1

0 0 0 0 0 0 0 0 1

0 0 0 0 0 0 0 0 0 1

0 0 0 0 0 0 0 0 0 0 1

0 0 0 0 0 0 0 0 0 0 0 1

0 0 0 0 0 0 0 0 0 0 0 0 1

0 0 0 0 0 0 0 0 0 0 0 0 0 1

0 0 0 0 0 0 0 0 0 0 0 0 0 0 1

0 0 0 0 0 0 0 0 0 0 0 0 0 0 0 1

fi te 15 15 te 16 16

va 0 te 15 15 te 16 16

ou dwls so sc
